# Identification of a Novel Nonsense Mutation in the *IGSF1* Gene Reveals Sex-Specific Phenotypic Variability Within a Single Family

**DOI:** 10.3390/children12121682

**Published:** 2025-12-11

**Authors:** Rosario Ruta, Nicoletta Massaccesi, Mafalda Mucciolo, Alessandro Sparaci, Enrica Fabbrizi, Antonio Novelli

**Affiliations:** 1 Translational Cytogenomics Research Unit, Bambino Gesù Children’s Hospital, IRCCS, Viale di San Paolo, 12, 00165 Rome, Italy; mafalda.mucciolo@opbg.net (M.M.); alessandro.sparaci@opbg.net (A.S.); antonio.novelli@opbg.net (A.N.); 2 Pediatric Departmental Simple Operative Unit, Civitanova Marche Hospital, ASUR Marche Area Vasta n. 3, 62100 Macerata, Italy; nicoletta.massaccesi@sanita.marche.it (N.M.); enrica.fabbrizi@sanita.marche.it (E.F.)

**Keywords:** *IGSF1*, IGSF1-deficiency syndrome, short stature, growth hormone deficiency

## Abstract

**Background**: The immunoglobulin superfamily member 1 (*IGSF1*) gene encodes for a transmembrane glycoprotein involved in crucial processes such as growth, metabolism, and reproductive function. Loss-of-Function (LOF) mutations in the *IGSF1* gene have been reported to cause the X-linked IGSF1 deficiency syndrome, a rare genetic condition that primarily affects males, characterized by hypothyroidism, macroorchidism, delayed puberty, obesity, and infertility. **Case Report**: In this study, we identified a novel hemizygous nonsense *IGSF1* variant c.1989G>A (p.Trp663Ter) in a male patient who initially presented with growth impairment and growth hormone deficiency (GHD), with a positive family history on the maternal lineage. Notably, the proband does not present with macroorchidism, a feature typically associated with IGSF1 deficiency. The variant was also found in his heterozygous sister, who presented with isolated growth hormone deficiency, and in his mother, who displayed hypertension and thyroid dysfunction but no significant growth impairment. **Discussion:** This phenotypic variability suggests a differential expression of IGSF1-related symptoms depending on zygosity and sex within the same family, probably explained by X-chromosome inactivation (XCI) in females, which can lead to varying degrees of functional IGSF1 expression in different tissues. **Conclusions:** This case highlights the intrafamilial phenotypic variability associated with *IGSF1* mutations, illustrating differences between male and female carriers and highlighting the importance of genetic testing in patients with similar clinical presentations.

## 1. Introduction

The Immunoglobulin superfamily member 1 (*IGSF1*) gene, located in the Xq26.1 region, encodes for a transmembrane glycoprotein [1]. It consists of 12 multiple immunoglobulin-like domains, which undergo co-translational cleavage during its synthesis, resulting in the expression of only its seven C-terminal immunoglobulin-like domains on the extracellular side of the plasma membrane [2]. The *IGSF1* gene is primarily expressed in the brain, pituitary gland, and testis, suggesting a significant role in neuroendocrine function and cellular interactions [3]. The primary function of IGSF1 is the regulation of gonadotropin-releasing hormone (GnRH) secretion, which is crucial for processes such as growth, metabolism, and reproductive function [4]. IGSF1 protein facilitates cell–cell interactions and may contribute to the maintenance of neuronal networks involved in endocrine signaling [5]. Experimental models have shown that IGSF1 plays a key regulatory role in TRH receptor (TRHR) trafficking and signaling, thereby influencing the sensitivity of thyrotropes to hypothalamic TRH stimulation [6]. Moreover, IGSF1 deficiency has been associated with somatotrope hyperfunction, with increased GH secretion and elevated IGF-1 levels in both humans and mice, suggesting a broader role in pituitary cell regulation [7].

Loss-of-function (LOF) mutations in the *IGSF1* gene have been described in the X-linked IGSF1 deficiency syndrome [8,9], a rare genetic condition characterized by central hypothyroidism, macroorchidism, variable prolactin and growth hormone levels, delayed puberty, obesity, and infertility, which can manifest in a range of clinical phenotypes [10]. Existing literature suggests that males with hemizygous loss-of-function mutations often exhibit a more severe phenotype compared to heterozygous females, who may experience milder manifestations or present with distinct endocrine abnormalities [11].

Here, we describe the case of a boy with growth impairment and growth hormone deficiency, who has a family history of short stature on the maternal lineage. Genetic analysis revealed a novel nonsense hemizygous variant in the *IGSF1* gene, which was also found in the mother and sister, both exhibiting mild symptoms. While this inheritance pattern is well established, our case provides novel insights that further expand the phenotypic spectrum of IGSF1 deficiency. In particular, we describe a male patient without macroorchidism, a frequently reported feature as a hallmark of the syndrome. Moreover, our findings highlight intrafamilial phenotypic variability among relatives carrying the same variant.

## 2. Case Report

An 11-year-old male patient came to our attention due to a significant reduction in statural growth. He was born at full term via cesarean section with a birth weight of 4.080 kg (97th percentile) and was classified as large for gestational age (LGA). He was the second child of non-consanguineous parents. The mother had a history of thyroid disease; however, no biochemical data (TSH, FT4, thyroid autoantibodies) were available to determine whether this indicated primary or central hypothyroidism; the patient’s older sister had been treated with growth hormone (GH) for growth hormone deficiency (GHD) from the ages of 10 to 15 years (Table 1).

Neonatal screening for congenital hypothyroidism (CH) was performed using TSH (thyroid-stimulating hormone) measurements on a Guthrie card, which was within the normal range. The patient’s early medical history was unremarkable, with no signs of neonatal jaundice, hypotonia, or failure to thrive. At 8 months of age, he underwent surgery for Meckel’s diverticulum following the onset of hematochezia. Psychomotor development followed typical patterns. Height and weight had been within normal limits until the age of 9 years, when a deceleration in statural growth was noted alongside weight gain.

Upon referral, the patient’s height was measured at 134 cm (−0.95 standard deviation score [SDS]), with a sitting height/height ratio of 0.56 (+2.5 SDS). His body mass index (BMI) was 23.9 kg/m^2^ (+1.54 SDS). Growth assessment revealed a marked reduction in growth velocity over two consecutive years. Between 9 and 10 years of age, the patient grew from 129 cm to 131.4 cm (2.4 cm/year), and from 10 to 11 years, growth velocity further declined to 2.6 cm/year (−3.66 SD). Tanner staging indicated pubic hair stage 1 (PH1) and genital stage 1 (G1), with testicular volume of 3 mL. Physical examination was otherwise normal, except for a single café-au-lait spot located on the left hemithorax. X-ray of the left hand revealed a bone age delay of 9 years and a curved radius. A comprehensive clinical and laboratory evaluation was performed to rule out pathological conditions known to impair growth. Celiac disease, cardiovascular diseases, pulmonary disorders, gastrointestinal and inflammatory bowel diseases, congenital or acquired intestinal abnormalities, malnutrition or low caloric intake, hepatic and hematologic disorders, renal diseases, juvenile rheumatoid arthritis, inborn errors of metabolism, central nervous system diseases, and skeletal dysplasias were all excluded based on clinical examination, auxological history, blood and urine tests, and specialist assessments. Thyroid function tests initially showed a serum TSH level of 3.04 µUI/mL (normal range for this age: 0.25–4) and a free T4 at the lower limit of normal (5.4 pg/mL; reference range: 5.4–12.6). Subsequent testing confirmed an inappropriately normal TSH (3.16 µUI/mL) alongside decreased free T4 levels (4.1 pg/mL), consistent with central hypothyroidism.

The patient’s IGF-1 level was 109 ng/mL (−1.71 SDS for age and sex). Growth hormone (GH) secretion was found to be insufficient (<8 ng/mL) in two separate stimulation tests performed one month apart: the clonidine test and the arginine test, which resulted in peak GH values of 0.266 ng/mL and 1.787 ng/mL at 120 min, respectively. Brain magnetic resonance imaging (MRI) revealed no abnormalities in the brain or hypothalamic-pituitary region.

The patient began growth hormone therapy at 11.2 years of age, with a starting dose of 0.024 mg/kg/day. Concurrently, levothyroxine supplementation was initiated at a dose of 1 µg/kg/day.

**Figure 1:**
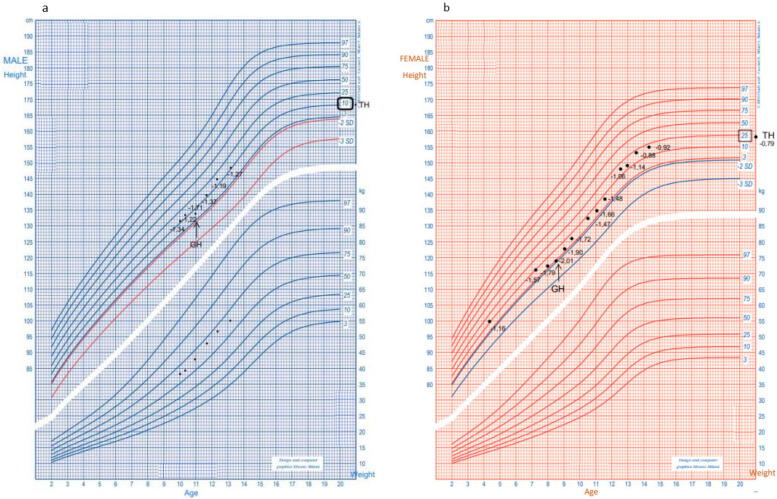
Growth charts of patients showing height and weight measurements taken at various time points before and after GH treatment. Panel (**a**) shows the proband, and panel (**b**) shows his sister.

Six months after the initiation of treatment, the patient exhibited a marked improvement in growth velocity (7.5 cm/year, +2.99 SDS) and demonstrated normal pubertal development (PH2, G2, with a testicular volume of 5 mL at 12.4 years). The dosages of both growth hormone and levothyroxine were gradually increased to accommodate the patient’s growth, with no significant adverse effects observed. The patient is currently 13.1 years old, with a height of 147.5 cm (−1.34 SDS), a BMI of 25.3 kg/m^2^ (+1.34 SDS), and a calculated growth velocity of 4.5 cm/year (−1.9 SDS) (Figure 1). His most recent serum TSH level was 0.07 µU/mL, with a free T4 level of 6.1 pg/mL.

The sister of the proband also exhibits short stature associated with growth hormone (GH) deficiency. Both siblings have been treated with GH; however, they display differing responses in terms of growth velocity, as illustrated by their respective growth charts (Figure 1).

**Figure 2:**
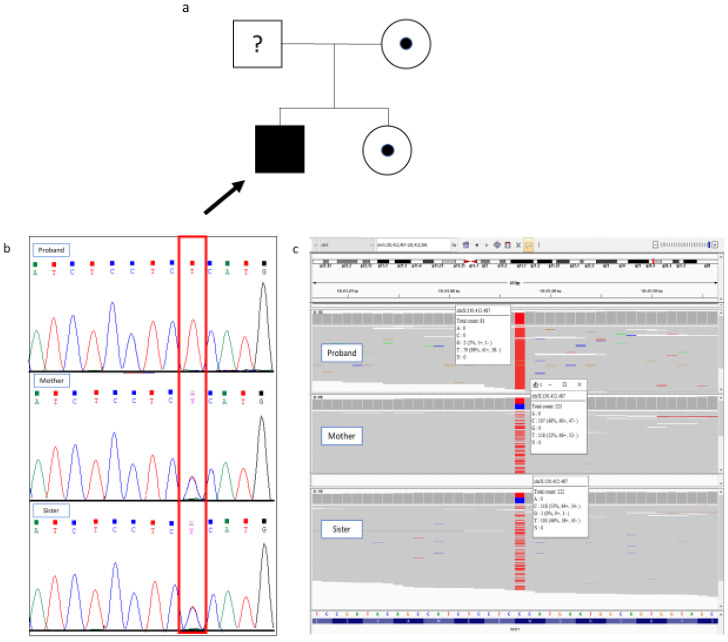
Genetic analysis of the family: (**a**) Pedigree of the family showing the affected proband, who is hemizygous for the variant and marked by an arrow, and the heterozygous carrier females, indicated by a black dot inside the symbol; the father was not available for genetic testing. (**b**) Sanger sequencing electropherograms confirming the presence of the c.1989G>A variant in the *IGSF1* gene. The red box highlights the presence of the variant in in the proband (hemizygous) and in his mother and sister (heterozygous carriers). (**c**) Integrative Genomics Viewer (IGV) visualization of the same variant as detected by next-generation sequencing (NGS), showing the aligned reads supporting the variant call.

The family history and clinical presentation in all three individuals of this study strongly suggested a genetic etiology, and further genetic testing was recommended. After written informed consent was provided, clinical exome sequencing (CES) was performed on the proband’s genomic DNA using ClinEX pro kit (4bases, Manno, Switzerland) on the NovaSeq6000 platform (Illumina, San Diego, CA, USA). In silico analysis was performed for coding regions and exon-intron junctions of the genes associated with Monogenic Short Stature. The reads were aligned to the human genome build GRCh37/UCSC hg19. Variant calling was performed with the Dragen Germline Enrichment application (Illumina, San Diego, CA, USA), while variant annotation and phenotype-based prioritization of candidate genes were carried out through the Geneyx Analysis software v6.0 (Geneyx Genomex, Herzliya, Israel). A minimum depth coverage of 30× was considered suitable for analysis. Exome sequencing data filtering was performed to identify protein-altering, putative rare recessive homozygous, compound heterozygous, and pathogenic or likely pathogenic heterozygous variants with an allele frequency < 1%. Variants were classified according to the American College of Medical Genetics and Genomics (ACMG) guidelines [12]. Next-generation sequencing (NGS) analysis detected a novel hemizygous variant in the *IGSF1* gene (c.1989G>A), resulting in a nonsense mutation p.Trp663Ter. The c.1989G>A variant is absent from population databases (gnomAD); it has not been described in the scientific literature, and it is not listed in reference databases. The pathogenicity of the identified nonsense variant was also evaluated using CADD (score = 37) and MutationTaster (prediction = “disease-causing”), both of which support a deleterious effect of the variant. Then, according to ACMG guidelines, it is classified as likely pathogenic (PM2, PVS1). Sanger sequencing was performed to validate the NGS result in the proband following a standard protocol (BigDye Terminator v3.1 Cycle Sequencing Kit, Life Technologies, Carlsbad, CA, USA). The segregation analysis of the variant was then performed on the mother and sister of the proband and showed that both were heterozygous carriers of the variant (Figure 2). This familial co-segregation further reinforces the pathogenic status by adding the ACMG PP1 criterion (co-segregation in the mother and sister), thus providing supporting evidence for this classification.

## 3. Discussion

The discovery of novel genetic variants associated with growth disorders in recent years has expanded our understanding of the molecular basis of endocrine diseases. The *IGSF1* gene encodes for a transmembrane protein involved in the regulation of growth hormone (GH) secretion by the pituitary gland and plays a significant role in the development and function of the hypothalamic–pituitary–gonadal (HPG) axis by influencing growth hormone secretion and thyroid hormone regulation [13]. LOF mutations in *IGSF1* result in defective protein function, leading to dysregulated GH secretion and growth impairment [6].

In this study, we identified a novel nonsense hemizygous *IGSF1* variant in a male patient who initially presented with growth impairment and growth hormone deficiency, with a positive family history on the maternal lineage. The genomic variant c.1989G>A in the *IGSF1* gene results in a change at the nucleotide level where a guanine is replaced by an adenine at cDNA position 1989. This nucleotide substitution leads to the creation of a premature termination codon (PTC) at the amino acid position 663 (total length of the wildtype protein 1341 amino acids), where tryptophan (Trp) is replaced by a stop codon (Ter), denoted as p.Trp663Ter. This PTC is expected to result in a truncated protein product due to the premature termination of translation, potentially leading to a loss of normal protein function through a mechanism such as nonsense-mediated mRNA decay (NMD) or the production of a nonfunctional protein. Moreover, the c.1989G>A variant is located in the carboxy-terminal domain of the protein, suggesting an important pathogenic role since most previously reported variants are located in this functional domain [3] (Table 2).

The key finding in this study is the novel nonsense mutation in *IGSF1*, which follows an X-linked recessive mode of inheritance. Interestingly, the affected male exhibited classic symptoms of IGSF1 deficiency, including growth impairment and growth hormone deficiency, but no macroorchidism, while his sister and mother, who were heterozygous for the same mutation, exhibited mild symptoms. The sister presented with isolated growth hormone deficiency without notable additional endocrine abnormalities. In contrast, the mother displayed mild hypothalamic–pituitary axis dysfunction, manifesting as hypertension and thyroid dysfunction, but no significant growth impairment. This phenotypic variability, particularly in the mother, suggests a differential expression of IGSF1-related symptoms depending on zygosity and sex. Additionally, the mother’s thyroid dysfunction may suggest a potential role of IGSF1 dysfunction in thyroid regulation, possibly modulated by her heterozygous status.

The treatment of GHD with growth hormone was successful in improving growth velocity in both children. However, the final height of the sister was lower than expected despite receiving treatment, suggesting that the *IGSF1* mutation may affect growth potential even with optimal GH therapy (Figure 1). This finding is important for clinicians, as it underscores the need for personalized treatment approaches in genetic forms of GHD.

As reported in the literature, in males with *IGSF1* mutations, GHD tends to be more severe, as they are hemizygous for the mutated allele. In contrast, females with heterozygous mutations often exhibit milder forms of the disease, with less severe growth impairment and hormonal abnormalities [11]. In addition, affected males tend to show delayed testosterone increase and growth spurt during puberty, but with normal testicular growth and the onset of macroorchidism in late adolescence [14]. In some cases, patients may present with normal testicular volume [15]. Other features include elevated BMI (also observed in female carriers) and slightly raised or high–normal adult IGF-1 levels. This differential phenotypic expression may be explained by X-chromosome inactivation (XCI) in females, which can lead to varying degrees of functional IGSF1 expression in different tissues [11]. This mosaicism can modulate the overall functional dosage of the affected gene, thereby influencing the severity and spectrum of clinical manifestations. In the context of the present family, differential XCI patterns represent a plausible explanation for the observed disparity between the mother and the sister, both of whom are heterozygous for the variant. A relatively balanced XCI pattern would be expected to mitigate the phenotype, whereas skewing toward inactivation of the wild-type allele could result in a more pronounced clinical presentation. While our data do not include direct assessment of XCI, the variability observed is consistent with the well-established impact of XCI mosaicism on expressivity in heterozygous females.

While the X-linked inheritance pattern of IGSF1 deficiency is well established, our case provides novel insights that further expand the phenotypic spectrum of this condition. In particular, we describe a male patient without macroorchidism, a feature that is frequently reported as a hallmark of the syndrome, highlighting that its absence does not exclude the diagnosis. This observation aligns with previous reports documenting variability in the onset and severity of endocrine manifestations, suggesting that additional genetic, epigenetic, or environmental factors may modulate the phenotype. Moreover, our findings highlight intrafamilial phenotypic variability among relatives carrying the same variant, reinforcing the need for careful family screening and individualized clinical assessment. Together, these observations emphasize that clinicians should consider IGSF1 deficiency even in the absence of classic features and that the phenotypic presentation can be broader than previously recognized.

This study has certain limitations, including the inability to perform additional genetic testing in other family members and the lack of functional testing on the investigated variant using appropriate cellular or animal models, which would further clarify its molecular mechanisms and contribution to the observed phenotype. However, the detailed phenotypic data we provide about the family add significant information to the existing literature on IGSF1-related endocrine disorders.

## 4. Conclusions

This case report highlights a novel loss-of-function mutation in the *IGSF1* gene in a family with growth hormone deficiency, growth impairment, and thyroid dysfunction. This case emphasizes the importance of genetic evaluation in endocrine disorders, and it enhances our understanding of the multifactorial nature of these conditions. The variability in phenotypic expression between male hemizygotes and female heterozygotes is an important aspect of this condition. Further studies are essential to explore the full spectrum of clinical manifestations associated with *IGSF1* mutations and to develop more targeted therapeutic strategies for affected individuals.

## Figures and Tables

**Table 1:** Clinical features of the proband, sister, and mother, providing a comparative overview of the key medical findings for each individual.

Clinical Features	Proband	Sister	Mother
GH deficiency	+	+	-
Thyroid dysfunction	-	-	+
Macroorchidism	-	/	/
BMI ^1^	elevated	normal	normal

^1^ BMI (body mass index).

**Table 2:** A comparison of previously reported IGSF1 pathogenic variants (NM_001555.5) and their associated phenotypes. This comparison illustrates the spectrum of clinical manifestations linked to IGSF1 deficiency. The novel nonsense variant identified in this study is reported in bold.

cDNA	Protein Change	Phenotype	Reference
c.1030C>T	p.Arg344Ter	Short stature	PubMed 38737102
c.1814G>A	p.Trp605Ter	Central hypothyroidism	PubMed 38295770
**c.1989G>A**	**p.Trp663Ter**	**IGSF1 deficiency syndrome**	**This study**
c.2008G>T	p.Glu670Ter	IGSF1 deficiency syndrome	PubMed 26840047
c.2303T>C	p.Leu768Pro	IGSF1 deficiency syndrome	PubMed 30086211
c.2380C>T	p.Arg794Ter	IGSF1 deficiency syndrome	PubMed 26840047
c.2548C>T	p.Arg850Ter	Central hypothyroidism	PubMed 38462462
c.2553T>G	p.Tyr 851Ter	Hypothyroidism	PubMed 31504637
c.2916G>A	p.Trp972Ter	Central hypothyroidism & testicular enlargement	PubMed 23143598
c.2974C>T	p.Arg992Ter	IGSF1 deficiency syndrome	PubMed 26840047
c.3034C>T	p.Gln1012Ter	IGSF1 deficiency syndrome	PubMed 26840047
c.3041G>A	p.Trp1014Ter	Central hypothyroidism	PubMed 38299175
c.3503G>A	p.Trp1168Ter	Central hypothyroidism & testicular enlargement	PubMed 23143598
c.3550C>T	p.Arg1184Ter	Central hypothyroidism	PubMed 23966245
c.3763C>T	p.Gln1255Ter	Central hypothyroidism	PubMed 32772515
c.3790C>T	p.Arg1264Ter	Central hypothyroidism	PubMed 31448769
c.3862C>T	p.Arg1288Ter	Hypogonadotropic hypogonadism & central hypothyroidism	PubMed 35431442
c.1750+1G>A	p.?	Central hypothyroidism	PubMed 38462462
c.2041+1G>A	p.?	Central hypothyroidism	PubMed 29425110
c.2608+1G>C	p.?	Short stature	PubMed 38737102
c.2609-1G>A	p.?	Central hypothyroidism	PubMed 38462462
c.3752-1G>A	p.?	IGSF1 deficiency syndrome	PubMed 26840047
c.1137_1138del	p.(Asn380GlnfsTer6)	Central hypothyroidism	PubMed 26302767
c.1705del	p.(Leu569PhefsTer11)	IGSF1 deficiency syndrome	PubMed 26840047
c.2233del	p.(Glu745LysfsTer28)	Central hypothyroidism & testicular enlargement	PubMed 23143598
c.2416_2417del	p.(Met806GlufsTer10)	Central hypothyroidism	PubMed 36464600
c.2896+1del	p.?	Central hypothyroidism & testicular enlargement	PubMed 35753512
c.3396_3397del	p.(Tyr1132Ter)	IGSF1 deficiency syndrome	PubMed 26840047
c.3518del	p.(Phe1173SerfsTer3)	Central hypothyroidism	PubMed 29662269
c.2284_2285insA	p.(Ser762TyrfsTer2)	Central hypothyroidism	PubMed 27310681
c.2407dup	p.(His803ProfsTer14)	IGSF1 deficiency syndrome	PubMed 26840047
c.2485dup	p.(Ala829GlyfsTer15)	Central hypothyroidism	PubMed 35350016
c.3251dup	p.(Gly1085TrpfsTer34)	Central hypothyroidism	PubMed 23363888
c.3581dup	p.(Glu1195ArgfsTer3)	Central hypothyroidism & testicular enlargement	PubMed 23143598
c.3017delinsTT	p.(Gly1006ValfsTer14)	IGSF1 deficiency syndrome	PubMed 26840047

## Data Availability

The original contributions presented in this study are included in the article. Further inquiries can be directed to the corresponding author.

## Abbreviations

The following abbreviations are used in this manuscript:

IGSF1	Immunoglobulin superfamily member 1
GnRH	Gonadotropin-releasing hormone
LOF	Loss-of-function
LGA	Large for gestational age
GH	Growth hormone
GHD	Growth hormone deficiency
CH	Congenital hypothyroidism
SDS	Standard deviation score
TSH	Thyroid-stimulating hormone
PH1	Pubic hair stage 1
G1	Genital stage 1
PH2	Pubic hair stage 2
G2	Genital stage 2
CES	Clinical exome sequencing
NGS	Next-generation sequencing
IGV	Integrative Genomics Viewer
HPG	Hypothalamic–pituitary–gonadal
PTC	Premature termination codon
NMD	Nonsense-mediated mRNA decay
XCI	X-chromosome inacrivation
